# La réparation sphinctérienne directe: points techniques, indications et résultats

**DOI:** 10.11604/pamj.2013.14.11.2024

**Published:** 2013-01-07

**Authors:** Said Ait Laalim, Abdelmalek Hrora, Mohammed Raiss, Karim Ibnmejdoub, Imane Toughai, Mohammed Ahallat, Khalid Mazaz

**Affiliations:** 1 Département de chirurgie générale (B), CHU Hassan II, Fès, Maroc; 2 Département de chirurgie générale (C), CHU Ibn Sina, Rabat, Maroc

**Keywords:** Incontinence anale, lésion sphinctérienne, réparation sphinctérienne directe, anal incontinence, Sphincter injury, direct sphincter repair

## Abstract

L'incontinence anale est un handicap physique, psychique et social majeur qui a de nombreuses causes différentes. Les méthodes actuellement disponibles pour améliorer les symptômes de cette incontinence sont les méthodes médicales et de rééducation d'une part et les méthodes chirurgicales d'autre part. Quatre techniques chirurgicales répondent à ces objectifs pour la plupart des malades: la sphinctérorraphie, la neuromodulation des racines sacrées, et les deux techniques de substitution que sont le sphincter artificiel et la graciloplastie dynamisée. La réparation sphinctérienne directe est la technique la plus utilisée dans le traitement chirurgical de l'incontinence anale (IA) par lésion sphinctérienne. Cette technique est envisageable chez les malades ayant une incontinence fécale en rapport avec des lésions limitées du sphincter anal externe. La technique chirurgicale est simple (myorraphie par suture directe ou en paletot) et bien codifiée. Les résultats fonctionnels sont imparfaits et se dégradent avec la durée du suivi. Une continence parfaite après réparation sphinctérienne est rarement acquise de façon durable: le malade candidat à cette approche thérapeutique doit en être averti.

## Introduction

L'incontinence anale (IA) est définie par l'émission involontaire et répétée du contenu rectal survenant chez un sujet âgé de plus de trois ans et évoluant depuis plus d'un mois [1]. Elle provoque un handicap à la fois physique, psychologique, social et professionnel, faisant appréhender au malade chaque acte de la vie quotidienne. La cause de l'incontinence fécale est souvent multifactorielle, mais les défauts anatomiques du sphincter contribuer à la maladie chez de nombreux patients. Les méthodes médicamenteuses et de rééducation sont fréquemment proposées comme traitement de première intention et environ un tiers des malades seront adressés pour une prise en charge chirurgicale sphinctérienne d'une incontinence fécale. Quatre techniques chirurgicales répondent à ces objectifs pour la plupart des malades: la sphinctérorraphie, la neuromodulation des racines sacrées, et les deux techniques de substitution que sont le sphincter artificiel et la graciloplastie dynamisée. Le chirurgien doit être compétent dans différents types de procédures et correspondre à la procédure avec les besoins du patient.

La réparation sphinctérienne directe est la technique la plus utilisée dans le traitement chirurgical de l'IA par lésion sphinctérienne. Ce serait l'objet de notre travail en mettant le point sur les techniques, les indications et les résultats de cette réparation sphinctérienne.

### Prévalence de l'incontinence anale

La prévalence de l'incontinence fécale est difficile à déterminer. Elle est fréquente, mais certainement sous estimée, cachée par le patient qui la vit comme un handicap honteux, et non recherchée par le médecin, souvent démuni devant sa prise en charge. L'analyse des différentes études épidémiologiques publiées entre 1992 et 2003 ont montré que le taux de prévalence de l'incontinence anale dans différents pays variait de 3 à 17%, jusqu' 30à 50% de la population gériatrique. Les grandes variations étant essentiellement dues à la définition de l'incontinence (excluant ou non la simple incontinence aux gaz), au caractère de la population étudiée et au mode de recueil des données.

Malgré le fait que le dommage obstétrical est une cause majeure de l'incontinence anale, deux études seulement effectuées auprès de la population générale montrent que la prévalence de cette incontinence anale était significativement plus élevée chez la femme que chez l'homme [2, 3]. Par contre toutes les autres études [4–9] ont rapporté des prévalences d'IA non différentes entre les hommes et les femmes. Il convient donc d'admettre que les données épidémiologiques infirment l'impression d'une prévalence de l'IA plus élevée chez la femme que chez l'homme et invitent donc à dépister l'IA trop souvent méconnue par les médecins et à faire un effort particulier auprès des patients masculins.

### Etiologies des lésions sphinctériennes

**Causes traumatiques:** Chez les femmes adultes, l'incontinence fécale est le plus souvent causée par un traumatisme obstétrical. Chez la primipare les lésions sphinctériennes sont présentent dans environ 35% des accouchements par voie basse, identifié par échographie endoanale, bien que certains femmes peuvent rester asymptomatiques [8]. Les lésions traumatiques du bassin peuvent aussi entraîner une incontinence anale par blessure du sphincter anal. Cela inclut les traumatismes associés à l'écrasement blessures du bassin, en particulier lorsqu'il est associé avec fracture du bassin, et les blessures associées à certaines formes d'agression sexuelle.

**Causes iatrogènes:** Les causes iatrogènes conduisant à l'incontinence anale par lésion sphinctérienne comprennent: La sphinctérotomie latérale, la fistulotomie, l'hémorroïdectomie et les manœuvres de dilatation anale.

### Quel bilan préopératoire? (Bilan des lésions sphinctériennes) Affirmer l'incontinence anale et évaluer sa sévérité

La sévérité de l'incontinence anale et de son retentissement peut être rapidement chiffrée par l'utilisation d'un score d'incontinence anale, dont il existe de nombreux exemples dans la littérature [10]. En pratique, c'est actuellement le score de la Cleveland Clinic (score de Jorge et Wexner) qui est le plus souvent utilisé dans les essais thérapeutiques actuellement publiés dans l'incontinence fécale (Tableau 1) [11].

**Tableau 1:** Calcul du score clinique d'incontinence anale

	Fréquence				
Type d'incontinence	Jamais	>1mois	< 1/mois	< 1/semaine	= 1/jour
Selles solides	0	1	2	3	4
Selles liquides	0	1	2	3	4
Gaz	0	1	2	3	4
Garniture	0	1	2	3	4
Altération de la qualité de vie	0	1	2	3	4

Le score d'incontinence anale s'obtient en additionnant les points obtenus en réponse aux questions posées.

Les notes 0 à 20 correspondent respectivement à une continence normale et à une incontinence anale maximale.

Les scores supérieurs à 10 et a fortiori à 15 correspondent à des incontinences sévères, habituellement traitées chirurgicalement.

Quant aux scores de qualité de vie, très nombreux également, ils ne peuvent être intéressants que dans le cadre d'étude, car ils sont d'utilisation difficile et rendent peu de service chez un patient donné, considéré isolément. Les outils les plus souvent utilisés dans l'évaluation de l'incontinence fécale sont le FIQL (Fecal Incontinence Quality of Life) [12] et le GIQLI (Gastrointestinal Quality of Life Index) [13]. Le premier concerne spécifiquement le domaine de l'incontinence fécale, le second celui des symptômes digestifs (questionnaire plus généraliste).

### Interrogatoire et examen clinique

L'interrogatoire doit insister chez le patient ou la patiente précisément sur ses antécédents proctologique-s (cure de fissure anale, de fistule anale, d'hémorroïdectomie), traumatiques (fracture du pelvis par exemple), agression sexuels et sur ses antécédents gynéco-obstétricaux (travail long, macrosomie, accouchement dystocique, épisiotomie voire déchirure périnéale) et recueillir le traitement réalisé à l'époque, ou ultérieurement. Il faut ensuite interroger des antécédents médicaux qui pourraient retentir sur la continence (existence d'un diabète ou d'une sclérose en plaques par exemple).

**Figure 1:**
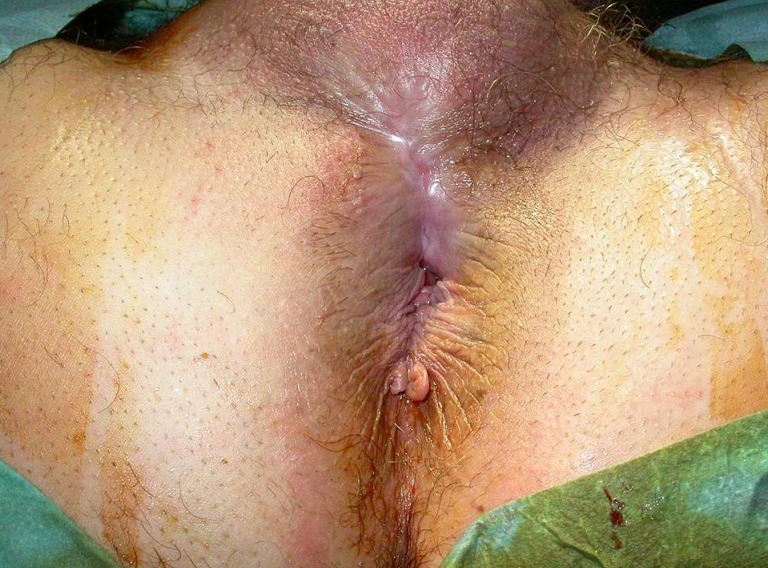
Cicatrise d'une lésion périnéale post traumatique. A noté l'absence de plis radiées en antérieur témoignant d'une lésion sphinctérienne à ce niveau

**Figure 2:**
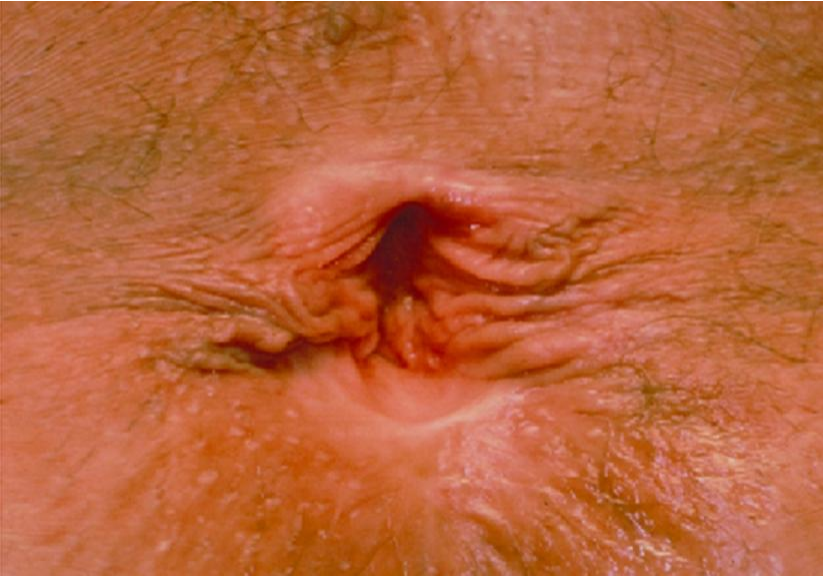
Cicatrice opératoire d'une fistulectomie dépourvue de plis radiés, témoignant d'une lésion sphinctérienne postérieure

L'examen clinique débute par une inspection qui doit permettre de déceler une béance anale spontanée, ou facile à la traction douce des plis. Une diminution de la distance ano-vulvaire ou une déformation de l'anus avec une absence localisée des plis radiés évocateurs d'une rupture sphinctérienne (rupture du même niveau). Des cicatrices périnéales: de la marge anale, épisiotomie para-sphinctérienne souvent difficiles à voir, en précisant le quadrant ou une suppuration anale chronique: Fissure, fistule (Figure 1, Figure 2)

Le toucher rectal est essentiel: il permet d'estimer la pression de base objectivée par le degré de résistance à la pénétration du doigt au repos et surtout la contraction volontaire, non seulement de l'appareil sphinctérien externe, mais aussi des muscles élévateurs. L'absence totale de contraction volontaire alors que l'anus est spontanément fermé doit faire évoquer une cause neurogène. La sensibilité de la peau périnéale doit être précisée également, car elle peut faire évoquer des troubles neurologiques plus complexes.

Cet examen clinique peut être mené chez un patient placé dans diverses positions. La position genu-pectorale offre la meilleure exposition sur la région périnéale, mais elle brime le sujet; la position gynécologique sur table spéciale permet une très bonne exploration de l'anus et du vagin et un palper bidigital de la cloison rectovaginale, pour estimer par exemple l'épaisseur des sphincters de l'anus en avant; la position de décubitus latéral est peut-être la moins bonne position, mais c'est la plus confortable pour le patient [14]. Un examen général et neurologique termine l'examen clinique.

## Bilan paraclinique

### Explorations fonctionnelles indispensables: 2 examens systématiques

**L**'**échographie endoanale (EEA)** est recommandée devant toutes IA, lorsqu'une origine sphinctérienne peut être suspectée et si une intervention chirurgicale pour réparation sphinctérienne peut être envisagée chez le patient [15]. L'EEA permet de rechercher ou de confirmer une lésion du sphincter anal interne et/ou externe et d'apprécier l'étendue de cette lésion qui conditionnera la chirurgie réparatrice à savoir qu'une lésion de plus de 90 degré sur la circonférence du sphincter peut difficilement être réparée par sphinctérorraphie (ce chiffre est variable selon les auteurs de 90° jusqu'à 180°) [16, 17]. De plus après réparation chirurgicale d'une lésion sphinctérienne, quand il persiste une IA, l'EEA permet de vérifier la qualité de la réparation [18–21].

**La manométrie anorectale** non invasive, permet de préciser le degré d'atteinte de la pression de repos et de la pression de contraction volontaire, (souvent déjà évoquée au doigt) la durée de cette contraction volontaire et la longueur du canal anal. Cet examen permet en outre d'évaluer la sensibilité et la compliance rectales. Elle permet d'obtenir des valeurs chiffrées, objectives, des pressions anales qui serviront de références en cas de traitement par biofeedback ou chirurgical (intérêt médico-légale); une étude rapporte la valeur péjorative de l'hypotonie anale quel que soit le traitement de l'IIA envisagé [22].

### Autres examens

La défécographie paraît utile lorsqu'un trouble de la statique rectale associé à l'incontinence est suspecté ou qu'il existe une constipation, que l'on explore également par un temps de transit colique [23].

Les explorations neuro-physiologique, intéressantes en recherche clinique, ne sont pas indispensable en pratique courante. Enfin un bilan uro-dynamique est à associer à l'évaluation digestive en cas de manifestations urinaires.

Au terme de ce bilan tant clinique que paraclinique, l'incontinence anale peut être classée selon l'atteinte de l'une ou de l'autre des composantes sphinctérienne. Dans les différentes causes d'incontinence anale, ces lésions s'associent à des degrés divers (rupture sphinctérienne et dénervation dans les lésions post-obstetricales par exemples). Cette classification est importante à avoir à l'esprit, car elle guide la décision opératoire et le choix de la technique de réparation; elle permet aussi d'en prédire et d'en évaluer les résultats.

## Réparation sphinctérienne directe: modalités thérapeutiques

C'est la technique la plus fréquemment réalisée dans le traitement chirurgicale de l'IA par déficit sphinctérien.

### Quelles préparations à l'intervention?

La prise en charge actuelle de la préparation des patients opérés d'une IA évolue progressivement vers une simplification du protocole. Le rasage périnéal doit être limité à la région opératoire. Pour la préparation intestinale, un régime pauvre en fibres pendant la semaine qui précède l'hospitalisation peut être suffisant et offrir un bon confort opératoire en toute sécurité. Une irrigation rectale à la Bétadine^®^, sur table, en position opératoire, peut compléter la préparation le jour de l'intervention. La préparation vaginale est discutée chez la femme: Ovules de Colpotrophine^®^ au cours du mois précédent l'intervention chez la femme âgée et irrigation vaginale abondante à la Bétadine^®^ la veille et le matin de l'intervention sont souvent effectués. Une antibioprophylaxie préopératoire est recommandée au cours de toute chirurgie réparatrice anale [24].

### Quelle modalité anesthésique?

Tout type d'anesthésie est possible: anesthésie générale, anesthésie locorégionale de type péridural ou rachianesthésie. Elle doit s'adapter à la durés opératoire habituellement comprise entre 60et 150minutes. L'infiltration à la xylocaïne^®^ adrénalinée de la zone opératoire est également utile. Elle améliore l'analgésie et le relâchement musculaire, limite le saignement et facilite la dissection. Enfin la réalisation d'un bloc pudendal par infiltration de xylocaïne ou de Naropeine dans les deux fosses ischiorectales en direction de la face interne des ischions (10à 20mm de chaque côté) permet de diminuer les douleurs au réveil [24].

### Position opératoire: Position gynecologique ou ventrale?

En France la position de référence est la position de la taille (ou gynecologique). Le patient est installé en décubitus dorsal, jambe fléchies sur le bassin et écartées, reposant dans des étriers. Le périnée doit être bien exposé, les fesses descendues au-delà de la table opératoire, l'appui se faisant sur le sacrum [25]. Les auteurs américains utilisent majoritairement la position ventrale (prone Jack-Knife position), le bassin reposant sur un rouleau de mousse et les fesses maintenues écartées par des bandes collantes [26]. Quelle que soit la position adoptée (celle-ci peut varier en fonction du type de procédure), il est de la responsabilité du chirurgien de veiller à la bonne installation du malade et d'éviter tout risque de lésions, notamment nerveuses, aux points de compression.

### Sondage urinaire

Le sondage urinaire est recommandé pour éviter les efforts de poussée abdominale et les rétentions d'urine à la période postopératoire initiale [26, 27]. Il facilite les soins locaux et évite les écoulements d'urines sur la plaie opératoire chez la femme. Le sondage est habituellement maintenu 2 à 3 jours.

### Quand envisager une colostomie de protection? De quel type?

La colostomie n'est pas justifiée de façon systématique dans la cure d'incontinence anale [18, 28]. Même en cas de procédure complexe d'implantation prothétique [29–32] la stomie peut souvent être évitée. Cependant, toutes les séries rapportent 20à 30% de patients stomisés [18, 33]. La décision de réaliser ou non une stomie est donc à adapter à chaque cas, en accord avec le patient. Si le type d'intervention n'est pas un critère décisionnel, le transit intestinal est en revanche un élément à prendre en compte. En cas de diarrhée importante et difficile à contrôler, il est préférable de dériver les matières pour éviter une reprise trop précoce du transit, susceptible de souiller les plaies opératoires [34]. De même, l'obésité et une trophicité périnéale médiocre, un diabète incitent à protéger les réparations sphinctériennes. Enfin les reprises chirurgicales après échec d'une première intervention sont également en faveur d'une dérivation intestinale [24].

La colostomie sigmoïdienne latérale sur baguette représente la meilleure option, assurant une dérivation de proche amont et complète du flux digestif. Elle est réalisée en fin d'intervention, volontiers sous cœlioscopie ou abord latéral, dans un site repéré en préopératoire, et immédiatement appareillée. La fermeture du bout distal est possible, mais on se prive alors de la possibilité de réaliser des tests de continence dans le sens du flux, par l'orifice distal de la stomie [24].

### Technique chirurgicale

Le siège de la rupture varie en fonction de l'étiologie et guide le geste opératoire.

### Sphinctérorraphie antérieure pour séquelles obstétricales

Les ruptures sphinctériennes obstétricales peuvent être dépistées dans les suites rapprochées de l'accouchement, après déchirure périnéale du troisième degré dont la réparation primaire s'est infectée et désunie. Le délai pour proposer une réparation est au minimum de 6mois. Il est indispensable que la cicatrisation anovulvaire soit acquise et que toute inflammation locale ait disparu avant d'entreprendre la réparation sphinctérienne [24].

Les ruptures sphinctériennes obstétricales peuvent être découvertes beaucoup plus tardivement: la rupture est initialement compensée par une trophicité musculaire suffisante notamment au niveau des muscles élévateurs, puis le relâchement tissulaire de la ménopause, l'accentuation de la descente périnéale avec l'âge démasquent les troubles de la continence [26, 28, 35]. Dans les cas des séquelles obstétricales, la rupture siège toujours sur l'hémi-circonférence antérieure du canal anal [18, 36]. Le défect sphinctérienne tend à s'accentuer avec le temps et les tractions musculaires de l'appareil sphinctérien annulaire.

### Voie d'abord et dissection du sphincter anal

La rupture sphinctérienne est habituellement facile à repérer: le coint périnéal postérieur a totalement disparu, faisant communiquer le vagin avec le canal anal, ou alors est remplacé par une bande cicatricielle, peu épaisse et non rétractile. Des fils de traction exposent cette zone. Une incision cutanée arciforme de 120° à 180° est menée sur la fourchette vulvaire en zone cicatricielle, et la plaie progressivement approfondie pour séparer et développer sur 4 à 6cm, deux lambeaux: l'un antérieur vaginal et l'autre postérieur ano-rectal. Cet abord est similaire à celui décrit pour la plicature rectale per voie périnéale dans la cure des réctocéles [37].

A partir du plan cutané cicatriciel, la cicatrice sphinctérienne, blanchâtre et scléreuse est repérée. La dissection se fait d'emblée vers la partie externe de la zone scléreuse correspondant au défect sphinctérien, pour deux raisons: La première est d'éviter toute effraction ou déchirure du canal anal par une dissection intempestive du tissu scléreux; La deuxième est que la dissection entre la muqueuse du canal anal et la sclérose est très difficile.

**Figure 3:**
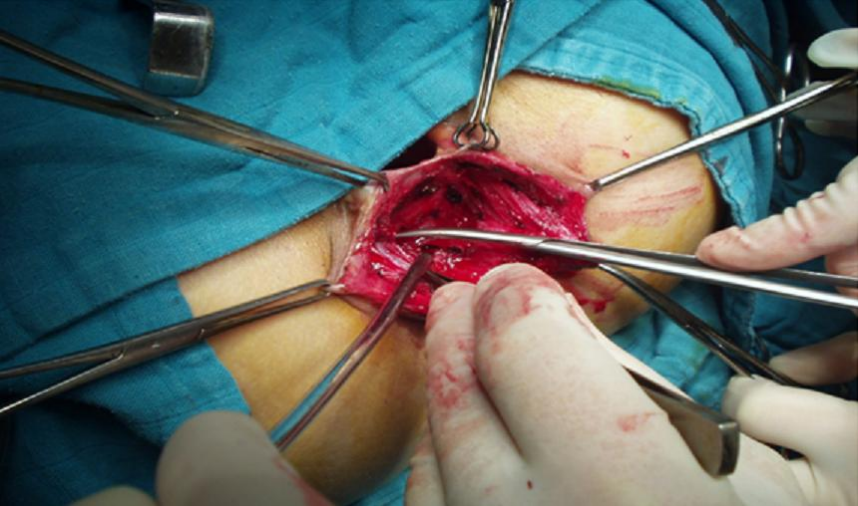
Dissection au ciseau du sphincter externe et du muscle releveur antérieur de l'anus

La dissection est poursuivie latéralement pour retrouver des extrémités musculaires, toujours proches de l'anoderme, qu'on dégage progressivement en prenant garde aux éléments vasculo-nerveux rejoignant le sphincter sur ses faces postéro-latérales. Cette dissection se fait aux ciseaux fins et au bistouri électrique, ce qui déclenche des contractions utiles au repérage musculaire. Elle doit dégager le sphincter dans toute son épaisseur en servant d'un fil tracteur pour exposer ses différentes faces [24] (Figure 3).

Certains essayent de repérer et séparer sphincters interne et externe pour en faire une réparation distincte [26]. D'autres au contraire recommandent d'éviter cette manœuvre [27, 36, 38] considérant que le sphincter interne est trop peu épais et trop fragile pour pouvoir être réparé de façon indépendante.

Une fois la face externe du sphincter externe libérée sur environ un quart de la circonférence, il faut cliver sa face inférieure de la région sous cutanée, puis pousser la dissection sur toute sa hauteur jusqu'à environ trois centimètres. Cette libération doit être suffisante pour permettre une suture correcte de ses extrémités. Il faut disséquer le sphincter (et sa cicatrice) sur toute sa hauteur et retrouver latéralement ses segments sains.

### Myorraphie des élévateurs de l'anus (Figure 4, Figure 5)

Avant la réparation sphinctérienne proprement dite, les deux faisceaux des élévateurs de l'anus, saisis très en arrière par deux pinces d'ombrédanne dans les angles de l'incision, sont rapprochés par deux points (Prolène ou Vicryl00), en prenant garde à les placer très en arrière pour ne pas rétrécir le vagin. Ce geste corrige un diastasis lévatorien fréquents dans les séquelles obstétricales, et participe à l'allongement du canal anal [26, 36].

**Figure 4:**
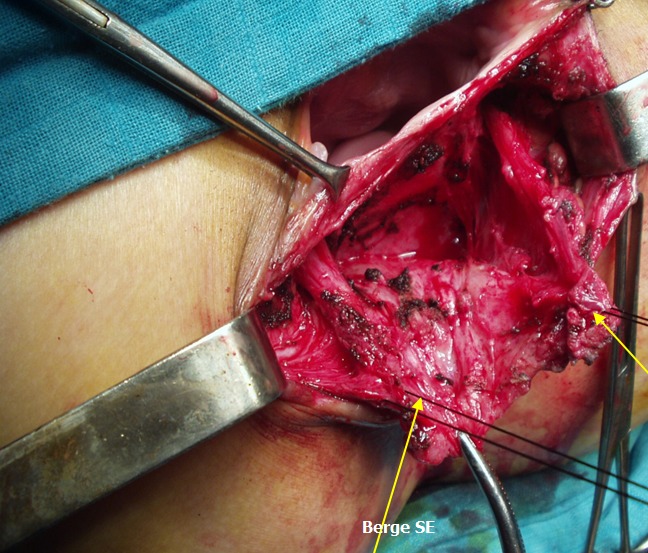
Image per-opératoire montrant, la dissection des berges du sphincter externe et du muscle releveur de l'anus

**Figure 5:**
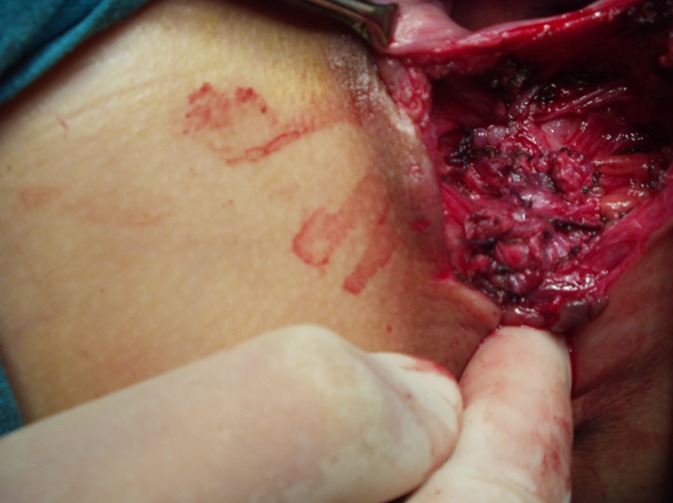
Transposition antérieure du muscle releveur de

### Reconstitution du canal anal

La reconstitution de l'anneau sphinctérien permet de reformer la zone cutanée sensible du canal anal et la ligne pectinée. Trois à six points de Vicryl 000 sont placés sur la zone cutanéomuqueuse dégagée du sphincter, en débutant par le point le plus médian. Un fil long est laissé sur ce qui correspond à la mage anale reconstruite. En cas de suture muqueuse importante, il est sans doute prudent de protéger la réparation par une colostomie, car la désunion de cette zone expose à l'infection et au lâchage du montage [24].

### Réparation directe (Figure 6)

On peut faire une réparation directe par apposition simple des deux fragments de sphincter, par l'intermédiaire des moignons fibreux. La cicatrice fibreuse correspondant à la rétraction du sphincter est pour certains auteurs réséquée, pour d'autres, préservée pour appuyer les points de suture.

**Figure 6:**
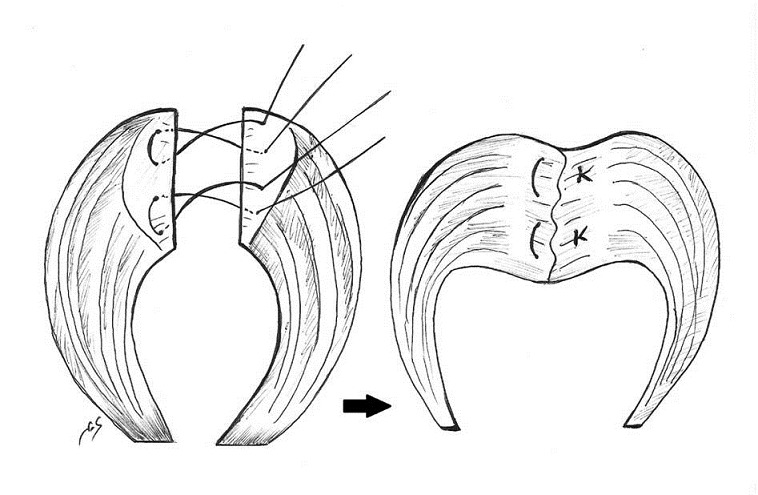
Schéma illustrant la sphinctérorraphie directe

### Réparation en paletot (overlapping technique) (Figure 7)

Les deux fils de traction exposent les extrémités sphinctériennes et l'on vérifie, en les croisant, qu'elles ont été suffisamment mobilisées pour obtenir un paletot de 2cm environ [18]. Avant la suture, il est nécessaire de calibrer l'occlusion anale crée par la réparation. Le montage doit serrer le cinquième doigt [39, 40] et/ou admettre une bougie de 15mm [27]. La première rangée verticale de points séparés prend appui sur la face externe du moignon fibreux d'amont et sur la face interne du moignon fibreux d'aval. La deuxième rangée verticale de points séparés plaque le moignon fibreux d'aval sur le moignon fibreux d'amont, les fils utilisés sont plutôt non résorbables.

**Figure 7:**
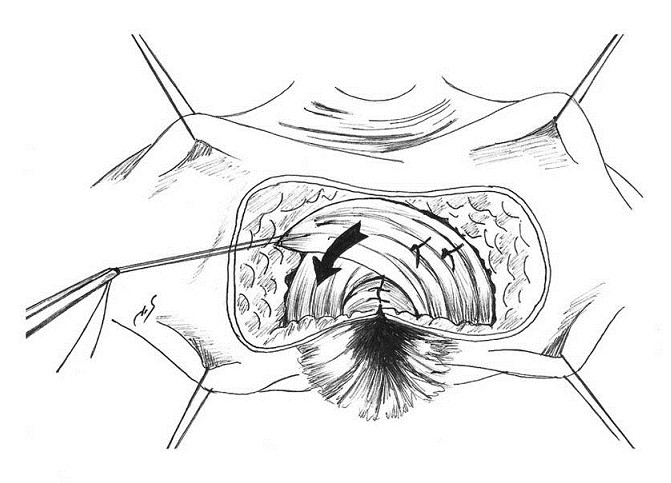
Schéma illustrant la sphinctérorraphie dite en paletot

### Fermeture cutanée

En fin de réparation, la plaie est largement irriguée à la Bétadine diluée. La fermeture cutanée ne doit pas être hermétique. Les plans sous-cutanés sont rapprochés lâchement au fil résorbable. La plaie cutanée est partiellement refermée en T également au fil résorbable, ce qui permet d'allonger la distance anovulvaire et reconstitue la paroi postérieure du vagin. La partie centrale du T est laissée ouverte pour assurer le drainage de la plaie, éventuellement à l'aide d'une petite mèche retirée au deuxième jour postopératoire. D'autres proposent une fermeture complète et un drainage aspiratif de petit calibre conservé 48heures [24]. Il faut cependant prévenir le patient du fait que la suture cutanée sera lâche, voir non étanche, inesthétique initialement, et faite par des points séparés qui resteront longtemps en place, en raison des tractions qui s'exercent sur eux.

### Sphinctérorraphie pour séquelles traumatiques

Les séquelles traumatiques iatrogènes ou accidentelles peuvent intéresser n'importe quel quadrant de la circonférence anale. Les ruptures sont repérées cliniquement à l'aspect de la marge anale et de la cicatrice et à l'absence de plis radiés (en coup de hache), et échographiquement. La technique opératoire est identique, mais les conditions locales peuvent varier [41]: la sclérose souvent plus manquée que dans les ruptures obstétricales, rend le repérage des extrémités musculaires plus difficile; les pédicules vasculo-nerveux qui abordent le sphincter en arrière et latéralement peuvent être lésés au cours de la dissection qu'on limite à la sclérose en respectant les zones musculaires saines ; la suture en paletot s'appuie sur le tissu scléreux que l'on conserve.

### Quels soins postopératoires?

Chez les patients non stomisés, il est souhaitable que la reprise du transit ne soit pas trop rapide. De l'Imodium peut être prescrit, mais il peut rendre difficile l'obtention des premières selles. Passé 48heures, le transit est facilité par des fibres et de petits lavements évacuateurs dés la première sensation de besoin. Les laxatifs osmotiques sont parfois nécessaires. La sortie du patient est généralement autorisée entre le sixième et le dixième jour pour la plupart des interventions, lorsqu'un transit régulier s'est installé [24].

Les soins locaux comportent un nettoyage régulier des plaies opératoires à l'eau et à la Bétadine et un séchage soigneux, deux fois par jour et après chaque selle sous contrôle infirmier. Une vessie de glace peut limiter l'oedème local. La toilette à la douchette après le passage des selles semble être la méthode la moins agressive, le plus hygiénique et la plus antalgique. La prévention du risque thromboembolique est indispensable avec une mobilisation précoce du patient en lui demandant d'éviter de trop écarter les cuisses, notamment lorsqu'il s'assoit sur un siège de toilettes. La rééducation postopératoire est discutée. Elle peut, avec le traitement médical, améliorer le résultat de différents types de réparation [30, 42, 43]. Elle est à prescrire en cas de résultats imparfaits, lorsque tous les phénomènes douloureux postopératoires ont disparu et après un bilan manométrique de référence.

### Réparation sphinctérienne directe: indications

Les techniques de réparation sphinctérienne sont envisageables lorsqu'il existe des lésions du sphincter anal externe. Par convention, les techniques de réparation sphinctérienne sont applicables lorsque la perte de substance n'est pas trop étendue et en règle dans un champ inférieur à la moitié de la circonférence sphinctérienne. La zone devant faire l'objet d'une réparation est identifiée en préopératoire par les données de l'endosonographie.

Les indications les plus fréquentes de la réparation du sphincter anal externe concernent les malades souffrant de troubles de la continence après un accouchement. La réparation est envisagée immédiatement lorsque les lésions sphinctériennes sont identifiées c'est-à-dire dans les déchirures périnéales de degrés trois et quatre. La réparation peut être proposée beaucoup plus tardivement lorsqu'un défect sphinctérien (a posteriori post-obstétrical) est identifié dans le cadre du bilan étiologique d'une incontinence fécale. Dans ces contextes, les troubles de la continence relèvent rarement d'un mécanisme sphinctérien exclusif. Ainsi, la moitié des parturientes qui souffrent de troubles de la continence n'ont pas de lésion sphinctérienne identifiée ce qui suggère que d'autres mécanismes (notamment neuropathiques) sont en cause [44]. Enfin, les troubles de la statique pelvienne et les signes d'atteinte neurologique périphérique sont fréquents chez les malades incontinents. La présence de signes associés ne doit donc pas représenter une contre-indication à la réalisation d'une réparation sphinctérienne justifiée par la présence d'un défect (à l'exception des malades ayant un prolapsus rectal extériorisé).

### Quel type de lésion sphinctérienne a réparée?

La réparation sphinctérienne est toujours indiquée en cas de rupture grave démontrée [14]. À ce sujet, il existe une polémique pour savoir si devant une rupture minime, il ne faudrait pas proposer une neuromodulation d'emblée. En faite une rupture minime ne correspond qu'à une section partielle du sphincter externe, d'ailleurs, elle est rarement évidente cliniquement et plutôt de diagnostic échographique: la première question à se poser est alors de savoir s'il s'agit réellement d'une rupture avec rétraction très partielle (il est parfois relaté des rétractions de 20°), ou s'il s'agit d'une simple cicatrice au sein du sphincter. Une rupture totale, c'est-à-dire sur toute la hauteur de l'appareil sphinctérien, s'accompagne d'une rétraction importante, puisque le sphincter est un muscle circonférentiel [45].

Une incontinence anale pourrait théoriquement survenir après rupture isolée du sphincter interne, mais cette occurrence est rare. Le sphincter anal interne ne fait habituellement pas l'objet d'un geste de réparation dont la technique est difficile (il s'agit d'un muscle fin et fragile) et les résultats chirurgicaux décevants, voire délétères sur la fonction anale dans un essai contrôlé [46]. Finalement, c'est l'examen qui prime: soit la rupture est importante et il faut proposer une réparation directe première, soit la rupture est peu évidente et la contraction volontaire sur le doigt très faible, et l'indication sera celle d'une neuromodulation [45].

### Qu'il est le moment idéal de la réparation sphinctérienne?

Elle l'est sans aucune discussion devant une rupture fraîche. Elle l'est à distance du traumatisme et lorsque la cicatrisation cutanée est achevée, lorsque le défect est conséquent.

La période propice à la réalisation d'un geste de réparation sphinctérienne constitue un argument actuellement plus polémique que scientifique. Chez les parturientes ayant un périnée complet, la réparation sphinctérienne est effectuée au décours immédiat de l'accouchement. Les résultats fonctionnels et anatomiques sont mauvais dans plusieurs travaux récents. Après le geste, près de la moitié d'entre elles décrit des troubles de la continence et les trois quarts ont une persistance de lésions échographiques des sphincters anaux [47–49]. Certains estiment donc que la qualité des tissus, l'oedème et l'importance des dilacérations devraient faire préférer un geste différé, effectué par un chirurgien spécialisé. Cette proposition n'est actuellement pas encore validée par des études contrôlées.

### Quelle technique opératoire: directe ou paletot?

Pour la qualité des résultats, le type de réparation, directe ou en paletot, n'intervient pas [50, 51]. Deux essais contrôlés randomisés récents soulignent l'absence de différence fonctionnelle entre les deux méthodes de réparation [50, 52]. Dans un essai, les troubles de l'exonération sont plus fréquents chez les malades traités par une suture en paletot [50].

## Résultats de la réparation sphinctérienne et facteurs prognostics

### Complications

Les complications liées au geste sont représentées par les douleurs postopératoires, la constitution d'hématomes et d'infections dans le lit opératoire, les troubles de l'exonération ou encore une désunion de la zone reconstituée.

### Résultats des sphinctérorraphies

L'évaluation des techniques de réparation sphinctérienne est établie sur les données d'études de cohorte, de celles résultant de l'analyse prospective de séries de cas et de celles des analyses contrôlées randomisées.

A court terme après l'intervention, ces techniques restaurent une continence normale ou acceptable chez 60 à 90 % des patients (Tableau 2) [18, 26, 27, 53–59]. Mais un peu moins de la moitié des malades sont totalement continents et un malade sur cinq environ garde une incontinence pour les selles.

**Tableau 2:** Sphinctérorraphies. Résultats des séries récentes (d'après Madoff)

Auteur	Nombre	Excellent (%)	Acceptable (%)	Médiocre (%)
Jacobs et al.,1990 [53]	30	83	17	0
Fleshman et al., 1990 [27]	55	72	22	6
Gibbs and Hooks, 1993 [54]	33	73	15	12
Londono-Schimmer et al., 1994 [55]	60	60	18	22
Engel et al., 1994 [18]	55	79	17	4
Oliveira et al., 1996 [26]	55	71	9	20
Nikiteas et al., 1996 [56]	42	67	14	19
Gilliland et al., 1998 [57]	100	60	19	21
Rasmussen et al., 1999 [59]	38	68	13	18
Buie et al., 2001 [58]	158	62	26	12

Les bons résultats fonctionnels s'accompagnent d'une amélioration manométrique des performances sphinctérienne: allongement de la zone de haute pression et accroissement des contractions volontaires [26, 27]. L'image échographique postopératoire est également corrélée au résultat: en cas d'échec persistent des défect traduisant une réparation incomplète ou déficiente [16, 18].

On dispose principalement d'études ouvertes ayant un suivi long après réparation sphinctérienne. Les résultats de la réparation sphinctérienne se dégradent avec la durée du suivi: 49 % des malades ont une incontinence pour les selles et seuls 28 % des malades ont une continence parfaite 40 mois après le geste [60]. Dans un autre travail analysant le bénéfice symptomatique en moyenne 69 mois après le geste de réparation, la moitié des malades traités ont des troubles de la continence des selles et seuls 14 % des malades sont parfaitement continents [61]. Après un suivi moyen de 77 mois, les résultats symptomatiques sont encore moins bons dans l'expérience d'un troisième centre: aucun malade n'est parfaitement continent, seuls 10 % ont une continence correcte pour les selles et près de la moitié des personnes ayant répondu au questionnaire portent des protections quotidiennement [62]. On peut donc estimer que plus de deux malades sur trois adressés aux chirurgiens pour une incontinence d'origine sphinctérienne ne bénéficient pas ou insuffisamment durablement d'une réparation sphinctérienne. Ces malades sont donc des candidats potentiels à un geste de substitution sphinctérienne.

### Facteurs Pronostic

Différents facteurs sont prédictifs du résultat postopératoire. Anatomiquement, le caractère localisé et isolé de la rupture, affirmé sur la clinique, mais surtout en EEA est essentiel. La zone de rupture ne doit pas excéder 160-180° de circonférence anale pour espérer une réparation efficace [45]. Une masse musculaire résiduelle suffisante et fonctionnelle doit être présente [45]. Certains travaux ont montré, dans ce contexte, une association significative entre la sévérité de l'incontinence, la présence et l'intensité des lésions sphinctériennes observées lors d'une endosonographie, la longueur fonctionnelle du canal anal et la qualité de la contraction volontaire avant et après un geste de réparation sphinctérienne [47].

Pour Chen, l'existence ou non d'une neuropathie pudendale ne jouerait pas sur les résultats de la réparation directe [63]. Pour d'autres auteurs [64, 65], l'augmentation des temps de latence est un facteur de mauvais pronostic du résultat des réparations sphinctériennes. Cependant les mauvais résultats immédiats ou précoces des réparations sphinctériennes sont principalement dus à une persistance de la rupture, cela a été parfaitement montré par l'écho-endoscopie [16, 47, 60–66]. En revanche, la détérioration progressive des résultats avec le temps est plus difficile à interpréter. Des résultats actuels vont dans le sens de publications récentes qui tentent à montrer qu'avec la qualité de la réparation, l'atteinte neurogène périphérique joue un rôle important dans la qualité des résultats après réparation sphinctérienne [57, 63]. Une neuropathie associée peut en effet influencer la qualité du résultat, mais ne contre-indique en aucun cas une tentative de réparation locale.

De même, le caractère classiquement péjoratif de l'âge, de l'ancienneté des symptômes d'incontinence avant la prise en charge chirurgicale, d'une première réparation sphinctérienne n'est pas confirmé dans plusieurs études récentes [18, 26, 67]. Les données de la manométrie préopératoire ne sont pas non plus corrélées avec le résultat opératoire [26, 32].

Sur le plan étiologique l'incontinence anale par traumatisme chez l'homme, l'incontinence anale par fistulotomie et enfin l'incontinence anale par rupture obstétricale chez la femme obèse de plus de 50 ans ayant une descente périnéale seraient des facteurs de mauvais pronostic d'une réparation directe [56]. Les résultats sont globalement meilleurs dans les incontinences postobstétricales [64, 68]. Une étude plus récente faisant l'objet de comparaison de la réparation sphinctérienne post-obstétricale et post opératoire, entre 2 groupes identiques de malades (excluant les hommes et incluant seulement les ruptures antérieures du sphincter), elle montrée que la réparation chirurgicale des ruptures sphinctériennes postopératoires donne de bons résultats immédiats persistant avec le temps [69]. Ces résultats vont dans le sens de publications récentes qui tentent à montrer qu'avec la qualité de la réparation, l'atteinte neurogéne périphérique joue un rôle important dans la qualité des résultats après réparation sphinctérienne [57, 63].

Enfin, il est possible de proposer une sphinctérorraphie chez un patient ayant déjà eu cette opération une première fois [70, 71].

## Points essentiels

- Chez les femmes adultes, l'incontinence fécale est le plus souvent causée par un traumatisme obstétrical
- La réparation sphinctérienne directe est la technique la plus utilisée dans le traitement chirurgical de l'IA par lésion sphinctérienne
- L'EEA et la manométrie endo-rectale sont deux examens indispensables avant toute réparation sphinctérienne
- La colostomie n'est pas justifiée de façon systématique dans la cure d'incontinence anale
- La réparation sphinctérienne est toujours indiquée en cas de rupture grave démontrée. Elle l'est sans aucune discussion devant une rupture fraîche ou à distance du traumatisme lorsque la cicatrisation cutanée est achevée
- Pour une bonne réparation efficace La zone de rupture ne doit pas excéder 160-180° de circonférence anale
- Pour la qualité des résultats, le type de réparation, directe ou en paletot, n'intervient pas
- Les résultats de la réparation sphinctérienne se dégradent avec la durée du suivi
- Les mauvais résultats immédiats ou précoces des réparations sphinctériennes sont principalement dus à une persistance de la rupture
- Une neuropathie associée peut influencer la qualité du résultat, mais ne contre-indique en aucun cas une tentative de réparation locale
